# GSK3β Regulates Differentiation and Growth Arrest in Glioblastoma

**DOI:** 10.1371/journal.pone.0007443

**Published:** 2009-10-13

**Authors:** Serdar Korur, Roland M. Huber, Balasubramanian Sivasankaran, Michael Petrich, Pier Morin, Brian A. Hemmings, Adrian Merlo, Maria Maddalena Lino

**Affiliations:** 1 Laboratory of Molecular Neuro-Oncology, University Hospital Basel, Basel, Switzerland; 2 Friedrich Miescher Institute for Biomedical Research, Basel, Switzerland; Dresden University of Technology, Germany

## Abstract

Cancers are driven by a population of cells with the stem cell properties of self-renewal and unlimited growth. As a subpopulation within the tumor mass, these cells are believed to constitute a tumor cell reservoir. Pathways controlling the renewal of normal stem cells are deregulated in cancer. The polycomb group gene Bmi1, which is required for neural stem cell self-renewal and also controls anti-oxidant defense in neurons, is upregulated in several cancers, including medulloblastoma. We have found that Bmi1 is consistently and highly expressed in GBM. Downregulation of Bmi1 by shRNAs induced a differentiation phenotype and reduced expression of the stem cell markers Sox2 and Nestin. Interestingly, expression of glycogen synthase kinase 3 beta (GSK3β), which was found to be consistently expressed in primary GBM, also declined. This suggests a functional link between Bmi1 and GSK3β. Interference with GSK3β activity by siRNA, the specific inhibitor SB216763, or lithium chloride (LiCl) induced tumor cell differentiation. In addition, tumor cell apoptosis was enhanced, the formation of neurospheres was impaired, and clonogenicity reduced in a dose-dependent manner. GBM cell lines consist mainly of CD133-negative (CD133-) cells. Interestingly, *ex vivo* cells from primary tumor biopsies allowed the identification of a CD133- subpopulation of cells that express stem cell markers and are depleted by inactivation of GSK3β. Drugs that inhibit GSK3, including the psychiatric drug LiCl, may deplete the GBM stem cell reservoir independently of CD133 status.

## Introduction

Recent studies suggest that cancer stem cells are the driving force behind tumorigenesis [1]. CD133 (also known as Prominin 1) was identified as a surface marker of cancer stem cells in brain tumors [2]. As few as 100 CD133-positive (CD133+) cells were shown to induce tumors in transplantation experiments giving rise to a phenocopy of the initial neoplasia [2], [3]. CD133+ cells, which express multi-drug resistance and DNA repair proteins [4], are highly resistant to chemo- and radiation therapy. However, stemness is not restricted to the expression of the CD133 marker, since CD133-negative (CD133−) cell populations were also found to be tumorigenic [5]. Cancer stem cells have also been detected in glioblastoma (GBM), the most malignant human brain tumor, with an annual incidence of 36 per million and a mean survival of less than 1 year [6]–[8]. GBM, a highly invasive and proliferative tumor, manifests itself as a *de novo* lesion or progresses from less undifferentiated low-grade astrocytoma.

Bmi1 is a member of the polycomb group of proteins involved in brain development [9]. Polycomb group proteins maintain embryonic and adult stem cells by forming multi-protein complexes that function as transcription repressors [10]–[17]. Bmi1 is also involved in cancer by cooperation with Myc in lymphoma formation [18] and blocking of senescence in immortalized mouse embryonic fibroblasts through repression of the Ink4a/Arf-locus [19]. It is also amplified and/or overexpressed in non-small-cell lung cancer, colorectal carcinoma, nasopharyngeal carcinoma, medulloblastoma, lymphoma, multiple myeloma and primary neuroblastoma [9], [13], [19]–[22]. Whether Bmi1 is expressed in GBM is controversial [9]. In a mouse glioma model, Bmi1 was implicated in tumorigenesis in an Ink4a/Arf-independent manner [23]. Furthermore, it was shown recently that microRNA-128 inhibits proliferation and self-renewal in glioma at least partially by downregulating Bmi1 [24].

Glycogen synthase kinase 3 (GSK3), a serine/threonine kinase, regulates numerous signaling pathways involved in cell cycle control, proliferation, differentiation and apoptosis [25], [26]. The mammalian isoforms GSK3α and GSK3β are functionally independent as GSK3α cannot rescue the embryonically lethal phenotype of GSK3β (−/−) mice [27]. GSK3 has been described as a pro-survival factor in pancreatic cancer [28] and as a pro-apoptotic factor in colorectal cancer [29] and is interconnected with several pathways and implicated in Alzheimer's disease [30], diabetes [31], bipolar disorder [32], and more recently cancer [33].

We have analyzed the role of GSK3 in malignant gliomas and its links to critical signaling proteins. Downregulation of Bmi1 reduced GSK3β levels and induced the differentiation of malignant glial cells. Direct inhibition of GSK3β by lithium chloride (LiCl), SB216763 and siRNA decreased Nestin and Sox2 levels and induced the cell differentiation markers CNPase, glial fibrillary acidic protein (GFAP) and β-tubulin III. In addition, LiCl and SB216763 depleted cancer stem cells grown as human GBM *ex vivo* cell cultures, induced differentiation and inhibited neurosphere formation. Thus, GSK3 may represent a novel therapeutic target for malignant gliomas.

## Materials and Methods

### Patients

Tumor samples obtained from patients during a neurosurgical procedure were immediately frozen and kept at −80°C. All patients gave their written consent for the neurosurgical procedure and for anonymous scientific analysis of diseased tissue according to the guidelines of the Ethics Committee of Basel, Switzerland (EKBB).

### Cell culture and reagents

LN319, LN18, LN215, U373, LN229, LN401, U343, U87, BS125 and Hs683 glioma cell lines with defined genetic status of *TP53, p16/p14* and *PTEN* [34], DAOY medulloblastoma and B104 neuroblastoma cell lines were cultured in Eagle medium supplemented with 25 mM glucose, glutamine, standard antibiotics, and 10% FCS. BS287 cells were cultured in Neurobasal medium (Invitrogen) supplemented with basic fibroblast growth factor (20 ng/ml, Invitrogen), epidermal growth factor (20 ng/ml, R&D Systems), B27 (1x) and N2 supplement (0.5x) (Invitrogen). All cells were maintained at 37°C in 5% CO_2_. The cell lines were seeded in 6-cm plates at 5’000–10’000 cells/cm^2^ and grown for 24 h prior to treatment. For cell counting, cells were treated for 72 h as described in the figure legends and counted by hemocytometer. Lithium chloride (LiCl) was obtained from MERCK and SB216763 from Tocris. Drug concentrations used are indicated in the figure legends. LiCl was dissolved in PBS and SB216763 in DMSO and stored at −20°C. The EGFR inhibitor AEE788 was provided by Novartis Pharma. The γ–secretase inhibitor DAPT was obtained from Roche.

### Colony formation assay

For each cell line, 500 cells were plated in triplicate into 94-mm Petri dishes containing 10 ml of culture medium with 10% FCS. Cells were grown for 14 days at 37°C and 5% CO_2_, during which period the medium was not changed. Cells were then fixed with 4% formaldehyde in 1x PBS and stained with crystal violet.

### BS287 “*ex vivo*” cell line formation and neurobasal medium

Following informed consent, a tumor sample classified as GBM based on the WHO criteria was obtained from a patient undergoing surgical treatment at the University Hospital, Basel, Switzerland. Within 1–3 h after surgical removal, the sample was treated with the Neural Tissue Dissociation Kit (Miltenyi Biotec GmbH) according to the manufacturer's protocol. Tumor cells were cultured in NBE media. Uncoated plastic dishes were used for neurosphere culture of NBE cells.

### Plasmids, lentiviruses and transfection

The lentiviral vectors pLKO.1-puro-scrambled-shRNA (Addgene) and pLKO.1-puro-shRNA (Sigma, sh1061: *CCGGCCTAATACTTTCCAGATTGATCTCGAGATCAATCTGG AAAGTATTAGGTTTTT*, sh693: *CCGGCCAGACCACTACTGAATATAACTCGAGTTATA TTCAGTAGTGGTCTGGTTTT*) targeting Bmi1 were transfected into HEK293 cells together with plasmids encoding the packaging (pCMV_dr8_91) and envelope proteins (pMD2-VSV-G) using CaCl_2_ precipitation. The concentration of infectious particles in the supernatant was titrated using HeLa cells. Glioma cells were transduced with infectious viral particles. Stably transfected clones were selected with 2 µg/ml puromycin. Bmi1 overexpression was obtained with pBABE-puro and pBABE puro-Bmi1 using CaCl_2_ precipitation for 8 h. Stably transfected clones were selected with 2 µg/ml puromycin. siRNA transfection for GSK3 was performed using the GSK-3α/β siRNA SignalSilence Kit (Cell Signaling Technology) according to the manufacturer's instructions. Cells were transfected using the Amaxa Nucleofector device (Lonza). Cells transfected with non-specific siRNA were used as a control.

### Transwell migration assays

Transwell migration assays were performed using modified Boyden chamber units with polycarbonate filters of 8-µm porosity (Costar). The lower side of the filter was coated with 10 mg/ml fibronectin for 2 h at 37°C. The bottom chamber was filled with DMEM containing 10% FCS. Cells (10^4^ per well in serum-free DMEM) were plated in the upper chamber and incubated for 24 h with or without GSK3β inhibitors. After removal of the remaining cells from the upper surface of the filter, migrated cells at the bottom of the filter were fixed with 3.7% formaldehyde in PBS and stained with 0.1% crystal violet. For each treatment, cells in 10 fields of view were counted in three independent experiments.

### Western blot analysis and antibodies

Cells were washed with 1x PBS, lysed in buffer containing 2% sodium dodecyl sulfate (SDS), 50 mM Tris pH 6.8, 0.1 M dithiothreitol (DTT), boiled and used either immediately or frozen at −20°C. Protein lysates were resolved on denaturing 8–12% SDS-polyacrylamide gels and transferred to nitrocellulose membranes (iBlot Gel transfer stacks, Invitrogen). The following primary antibodies were used: anti-Bmi1 (Upstate), anti-β-catenin and anti-Nestin (Santa Cruz Biotechnology); anti-β-tubulin III and anti-GFAP (Sigma); anti-CNPase (Chemicon); anti-GSK3β (Cell Signaling); anti-Notch2 (Developmental Studies Hybridoma Bank); anti-Sox2 (R&D systems); anti-CD133 (Miltenyi Biotec); anti-Akt and phospho-Akt (Ser-473) (Millipore, Billerica MA, USA), anti-p16/p14, anti-Bcl2, anti-Erk and anti-phospho-Erk (Santa Cruz Biotechnology, Santa Cruz CA, USA), anti-Actin (Sigma-Aldrich, St. Louis, USA). Decorated proteins were revealed using horseradish peroxidase-conjugated anti-mouse, anti-rabbit, anti-rat (New England Biolabs) or anti-goat (Pierce) secondary antibodies and visualized by the chemoluminescence detection system SuperSignal West Pico (Thermo Scientific). Protein bands were quantified with ImageJ software (http://rsb.info.nih.gov/ij/). Results were normalized to actin levels.

### Cell sorting and flow cytometry

Cell DNA content and apoptosis were analyzed by flow cytometry (CyAn ADP Analyzer, Beckman Coulter) and the results statistically evaluated with Summit v4.3 software. Cells were trypsinized, fixed in ice-cold 70% ethanol for 1 h and stained with 50 µg/ml propidium iodide for FACS analysis. Percent dead cells was determined from the proportion of cells in sub-G1 phase. Results are given as mean values from three independent experiments. For CD133 analysis, isolated cells were labeled with anti-CD133 antibody (1∶10) for 10 min at 4°C, washed with PBS and sorted (INFLUX Cell Sorter by BD Biosciences).

For BrdU analysis, cells were pulsed with 10 µM bromodeoxyuridine for 2 h and processed with the APC-BrdU kit according to the manufacturer's instructions (BD Pharmingen). For fluorescent labeling with GFAP, cells were fixed and permeabilized with the Cytofix/Cytoperm kit (BD Pharmingen). Permeabilized cells were incubated with anti-GFAP (1∶200) or matching isotype control antibody for 30 min on ice, washed twice and incubated with the corresponding secondary antibody for 30 min on ice and analyzed by flow cytometry (CyAn ADP Analyzer, Beckman Coulter).

### Immunocytochemistry

Cells were grown to 80% confluency as a monolayer as described above. Cells were then fixed in 4% PFA in PBS for 15 min at room temperature, washed with PBS and incubated with the primary antibody overnight at 4°C in PBS +1% BSA +0.1% Triton X100. After thorough washing with PBS, the secondary antibody was added for 3 h at room temperature. Cells were imaged by confocal microscopy. All tumor samples analyzed were stained with hematoxylin-eosin.

### Microarray analysis of glioma

BS series are primary tumor tissues obtained from patients diagnosed with primary CNS tumors classified according to the WHO grading system. Normal brain tissue used as a template for microarray was obtained from samples of brain surgery for non-neoplastic disease. Total RNA from two normal brains, 12 GBM and eight astrocytoma samples was amplified and labeled using the Affymetrix 2-cycle amplification protocol according to the manufacturer's instructions (Affymetrix). Samples were hybridized to Affymetrix U133v2.0 GeneChips and scanned using an Affymetrix Gene Chip scanner following the manufacturer's instructions. Expression values were estimated using the GC-RMA implementation in the Genedata Refiner 4.1 (Genedata, Basel, Switzerland) package. Data-mining and visualization was performed using the Genedata Analyst 4.1 package. All samples were quantile normalized and median scaled to correct for minor variation in expression distribution. All microarray data reported in the manuscript are in accordance with the MIAME guidelines.

## Results

### Bmi1 is overexpressed in GBM, oligodendroglioma and astrocytoma

**Figure 1:**
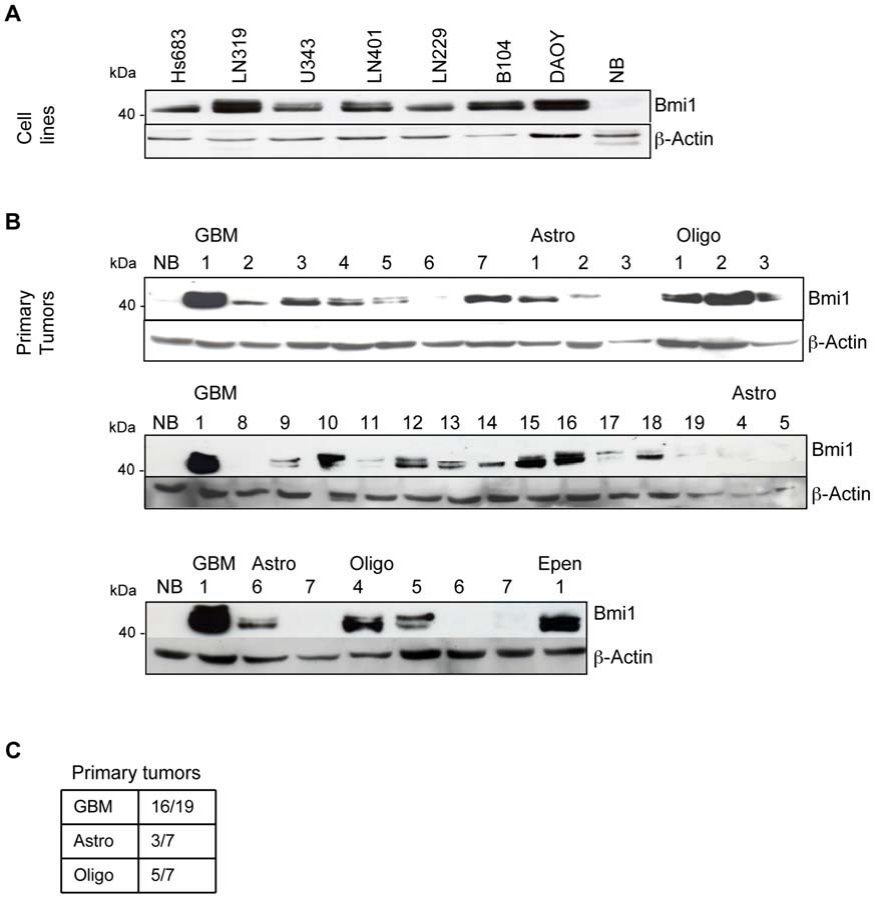
Bmi1 was highly expressed in GBM. Bmi1 expression in (A) GBM cell lines and (B) primary brain tumors. (C) Ratio of Bmi1-positive primary tumors. NB: normal brain. EPEN: Ependymoma.

Bmi1 overexpression has been reported in several different tumor types including medulloblastoma and neuroblastoma. In an analysis of Bmi1 mRNA and protein expression in GBM cell lines and primary brain tumors, all GBM cell lines expressed high Bmi1 levels, with the LN319 line having the highest expression comparable to the reference line DAOY [9] (Figure 1A and data not shown). In primary brain tumor samples, Bmi1 expression was marked in 16/19 (84%) of GBM, 5/7 (71%) of oligodendroglioma and 3/7 (42%) of astrocytoma. In contrast, fully differentiated normal brain tissue had no Bmi1 protein (Figure 1B, C).

### shRNAs against Bmi1 downregulates GSK3β

**Figure 2:**
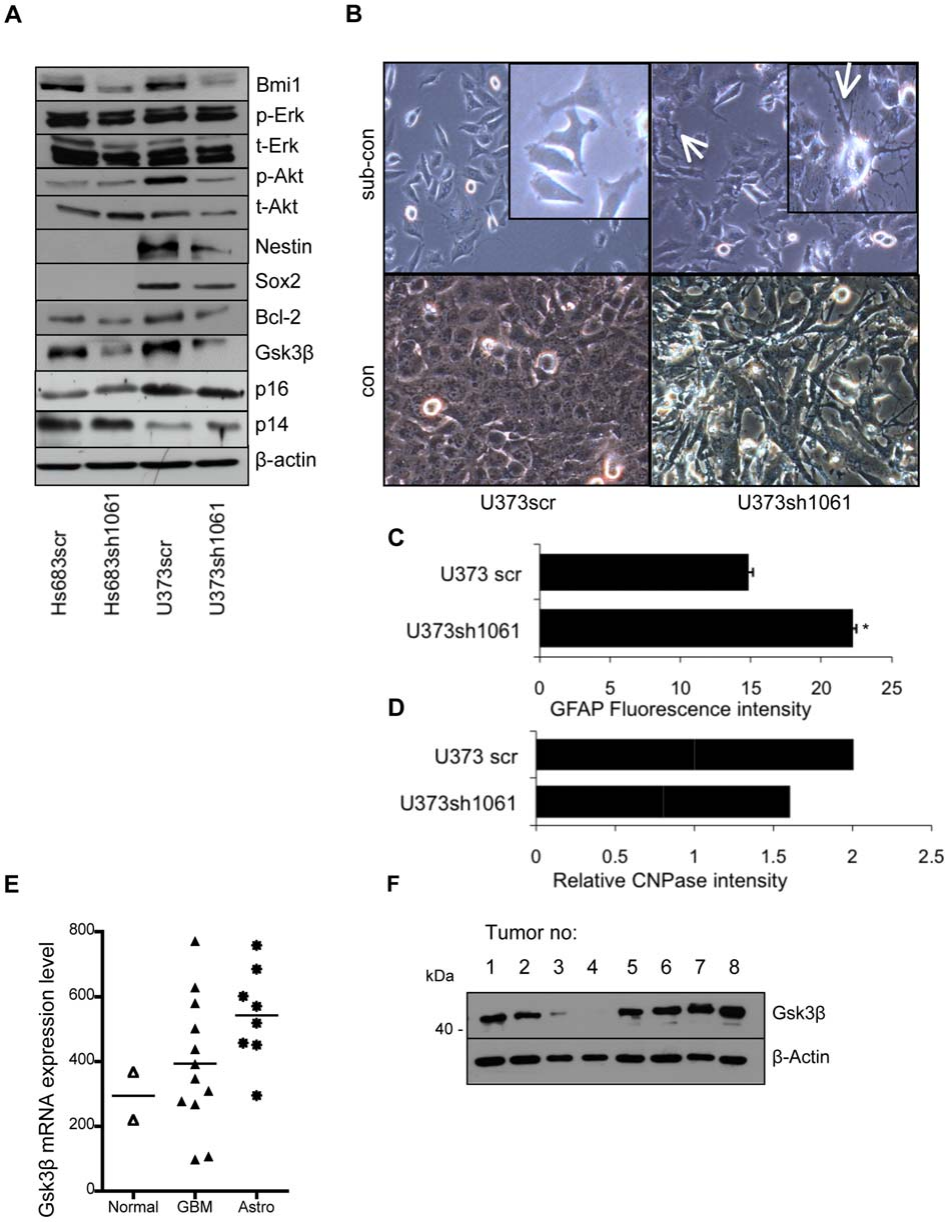
GSK3β was downregulated by reduction of Bmi1 expression. (A) GBM cell lines U373 and Hs683 transduced with shRNA against Bmi1 (sh1061) or with scrambled shRNA (scr). Bmi1 downregulation induced downregulation of the following proteins: p-AKT, Nestin (in U373), Bcl2, and GSK3β. No effects are evident on the p16/p14^ARF^, or p-ERK protein levels. (B) Scrambled control or U373 Bmi1-downregulated cells at subconfluency (sub-con) or confluency (con). Bmi1-downregulated cells show differentiated morphology (arrow: long, branched processes). (C) The glial fibrillary acidic protein (GFAP) levels in U373scr and U373sh1061 cells measured by flow cytometry. (D) Relative CNPase protein levels in U373scr and U373sh1061 measured by western blotting, quantified using ImageJ software and normalized to β-actin levels. (E) GSK3β mRNA values from a series of primary brain tumors (GBM and astrocytoma) and normal brain. (F) GSK3β protein is expressed in primary GBM. *P<0.05; one-way ANOVA (Newman-Keuls Multiple Comparison Test).

To study the role of Bmi1 in GBM, Bmi1 expression was knocked down using lentiviral-mediated delivery of shRNAs. Bmi1 was efficiently downregulated in the Hs683, U373, U87, and U343 GBM cell lines (Figures 2A and S1A and data not shown) using different shRNA sequences (Figure S1B). Since the Polycomb group gene Bmi1 is involved in the regulation of development and tumorigenesis, the effect of Bmi1 downregulation in GBM cells was screened by analysis of proteins involved in key cellular pathways of the cell cycle, development, metabolism, apoptosis and growth, including Erk, Akt, GSK3β, p16 and p14, Bcl-2, c-Myc, Nestin and Sox2. In contrast to non-neoplastic cells in Bmi1 knockout mice [19], Bmi1 downregulation in GBM cells did not affect Ink4a/Arf protein levels (Figure 2A). Bmi1 downregulation induced cell differentiation associated with morphological changes and decreased expression of the stem cell-related proteins Nestin and Sox2, accompanying induction of an astrocytic fate in U373 glioma cell line, determined by increased levels of the astrocyte-specific marker GFAP, and decreased levels of oligodendrocyte-specific marker CNPase (Figures 2A–D and S2B). In contrast, Bmi1 overexpression accompanied dedifferentiation as shown by increased Nestin expression (Figure S1C). Interestingly, GSK3β levels were markedly reduced (Figures 2A and S1A–B). This raised the question of whether GSK3β mediates the effects observed on cell differentiation. To this end, we used the small molecules LiCl and SB216763 as well as siRNA to interfere with GSK3β activity in GBM cells.

### GSK3β is expressed in GBM

Thirty-two primary tumor tissues obtained from patients diagnosed with GBM were studied using microarray and western blot analysis to measure GSK3β mRNA and protein levels. GSK3β mRNA were higher in GBM and astrocytoma patients compared with the control (Figures 2E, F and S2) and protein was found to be expressed in the majority of the tumors analyzed. The high GSK3β expression in astrocytoma was most probably due to greater necrosis in the GBM, with increased protein degradation (Figure S2B).

### siRNA- and drug-induced inhibition of GSK3 increases differentiation markers

**Figure 3:**
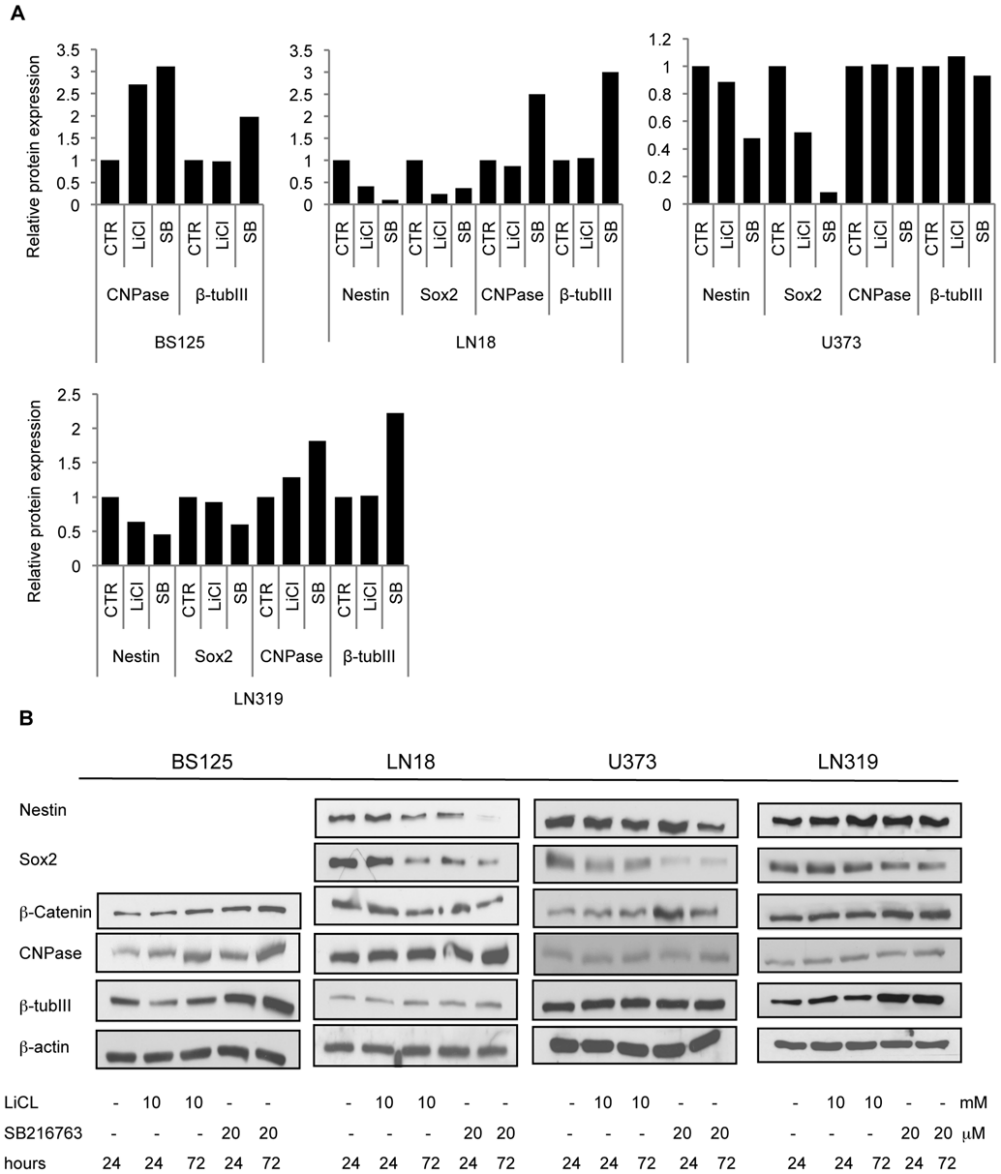
GSK3 inhibitors specifically induced differentiation in GBM cell lines. Western blot analysis of Nestin, Sox2, CNPase, β -tubulin III and β -actin (loading control) protein levels after treatment of GBM cell lines with GSK3 inhibitors (10 mM LiCl; 20 µM SB216763). Each cell line showed a pro-differentiation response to GSK3β inhibitor application. (A) Protein bands quantified with ImageJ software. Protein levels were normalized to β-actin for each cell line. (B) Corresponding western blots.

**Figure 4:**
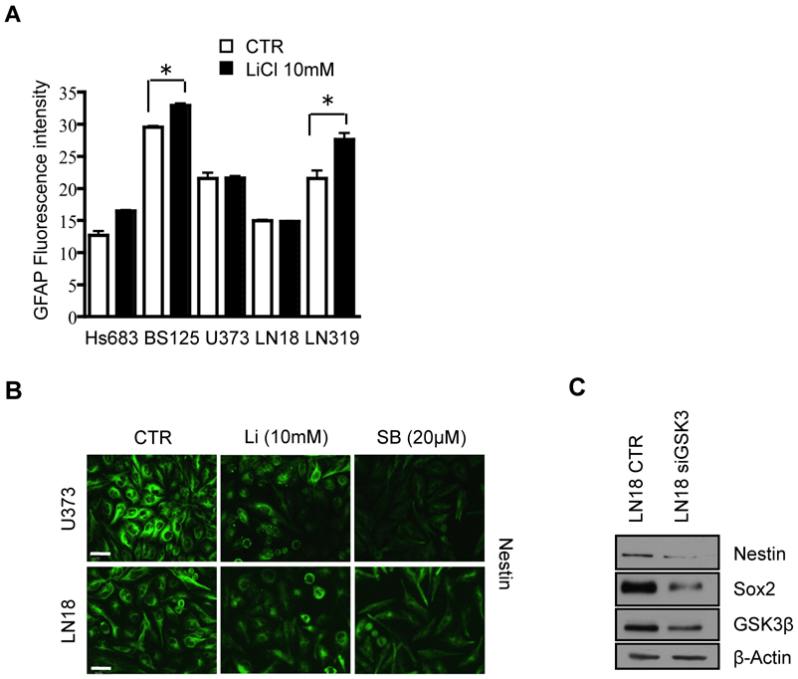
GSK3β downregulation by siRNA treatment induced differentiation. (A) The intensity of GFAP, an astrocyte-specific marker, was measured by FACS analysis of GBM cell lines treated with 10 mM LiCl for 72 h. (B) Nestin immunostaining of cell lines U373 and LN18 after GSK3β inhibition by LiCl (Li) or SB216763 (SB) for 72 h. (C) GSK3β siRNA reduced Sox2 and Nestin expression in the LN18 cell line. *P<0.05; one-way ANOVA (Newman-Keuls Multiple Comparison Test). Scale bar in B is 30 µm.

The effect of the inhibition of GSK3 on protein levels of progenitor (Nestin and Sox2) and differentiation (CNPase, GFAP, and β-tubulin III) markers in GBM cell lines U373, LN319, BS125 and LN18 was analyzed using the drugs LiCl and SB216763 or siRNAs. β-catenin is targeted for degradation upon phosphorylation by GSK3β [25]. Blocking GSK3β therefore leads to accumulation of β-catenin, which was used as a read-out for the effects of LiCl and SB216763 on GSK3 activity (Figure 3B). After 72 h treatment with LiCl and SB216773, Nestin protein level decreased in the Nestin-expressing U373, LN319 and LN18 cell lines (Figures 3A, B), which was confirmed by immunocytochemistry (Figure 4B). Nestin and Sox2 were not expressed in the “*ex vivo*” BS125 GBM cell line. Sox2 levels were reduced in LN18, U373 and LN319 upon GSK3 inhibition (Figure 3A, B). The oligodendrocyte specific marker 2′, 3′-cyclic nucleotide 3′-phosphodiesterase (CNPase) and the neuronal marker β-tubulin III increased in BS125, LN18 and LN319, (Figure 3A, B). GSK3 inhibition also increased the protein levels of the astrocytic lineage-specific marker GFAP in LN319 and BS125 (Figure 4A). Downregulation of GSK3 activity in LN18 by siRNA reduced Nestin and Sox2 protein levels (Figure 4C), confirming the specificity of the inhibitory drug SB216763 in blocking GSK3β. The effect of the specific GSK3 inhibitor SB216763 was more pronounced and consistent than the effect of LiCl, which is known to target other signaling molecules [26]. Thus GSK3 inhibition specifically decreased the expression of progenitor markers (Nestin and Sox2) and induced the expression of differentiation markers (neuronal marker β-tubulin III, oligodendrocyte-specific marker CNPase and the astrocytic marker GFAP) in a cell line-dependent manner.

### Inhibition of GSK3 depletes GBM cells with a stem cell signature

**Figure 5:**
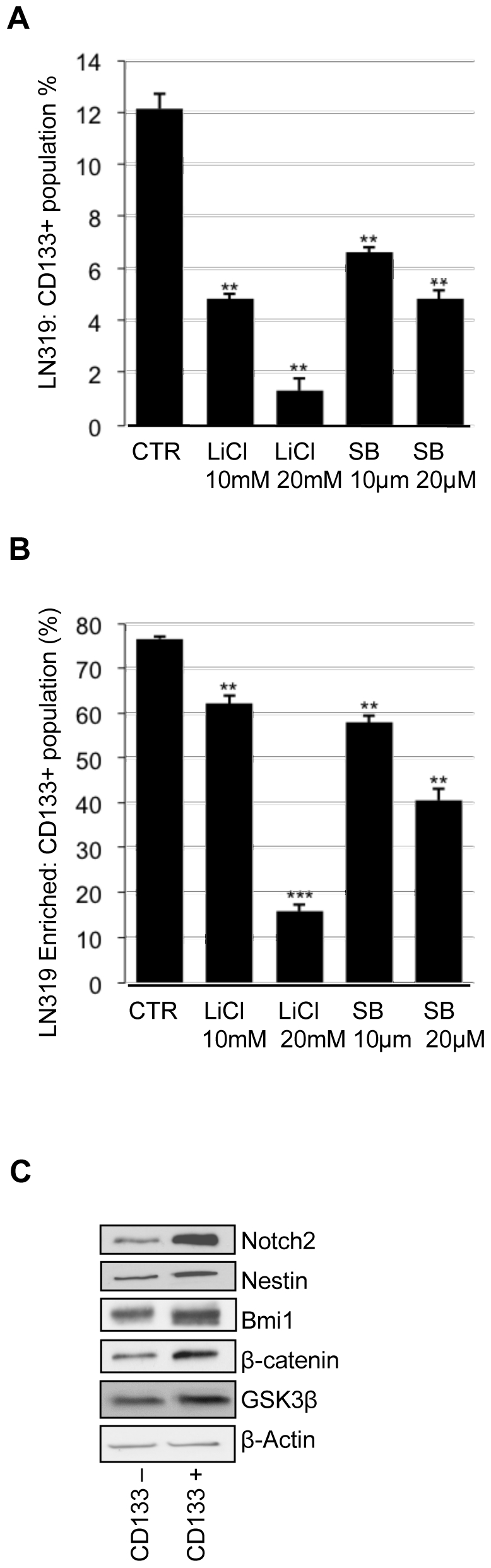
GSK3β inhibition reduced the CD133+ cell population of a GBM cell line. (A) LN319 GBM cell line was treated for 72 h with 10 or 20 mM LiCl or with SB216763 at 10 or 20 µM. GSK3β inhibition reduced the CD133-positive (CD133+) population. (B) LN319 CD133+ cells were enriched to 80% purity by cell sorting. The enriched population was treated for 72 h with 10 or 20 mM LiCl or with SB216763 at 10 or 20 µM. (C) Western blot analysis of Notch2, Nestin, Bmi1, β-catenin, GSK3β and β-actin in CD133+ and CD133-negative (CD133-) enriched populations compared with the control. **P<0.01; ***P<0.001; one-way ANOVA (Newman-Keuls Multiple Comparison Test).

The phenotypic switch towards differentiation in GBM cells following inhibition of GSK3 raises the question of whether GSK3 activity regulates cancer stem cell populations. CD133+ and CD133− cancer stem cells have been described in GBM [2], [3], [35]–[37]. In an analysis of eight different tumorigenic GBM cell lines (LN18, LN215, LN319, U373, LN229, U343, BS125 and Hs683) [34] for the presence of CD133+ cells, only LN319 contained CD133+ cells, at approximately 12%. To test whether the CD133+ population possessed a cancer stem cell-like character, the expression levels of stem cell markers were analyzed in the CD133-enriched population. Nestin, Notch2 and Bmi1 were highly expressed relative to the CD133− fraction (Figure 5C). GSK3β and β-catenin protein levels were also higher in the CD133+ population than in the control (Figure 5C). Inhibition of GSK3 in cell line LN319 with either LiCl or SB216763 showed a selective effect on the cancer stem cell-like population. LiCl at 10 mM and SB216763 at 20 µM induced a 50–60% depletion of CD133+ cells (Figure 5A). The effects of GSK3 inhibitory drugs were found to be specific in that the epidermal growth factor receptor inhibitor (AEE788) and the γ-secretase inhibitor (DAPT) did not significantly alter the CD133+ population (data not shown). To further consolidate this observation, we enriched the LN319 CD133+ population by FACS sorting, obtaining a CD133+ cell population of approximately 80%. This was maintained for several passages and then subjected to inhibition of GSK3 by LiCl or SB216763, which depleted the CD133+ fraction (Figure 5B).

**Figure 6:**
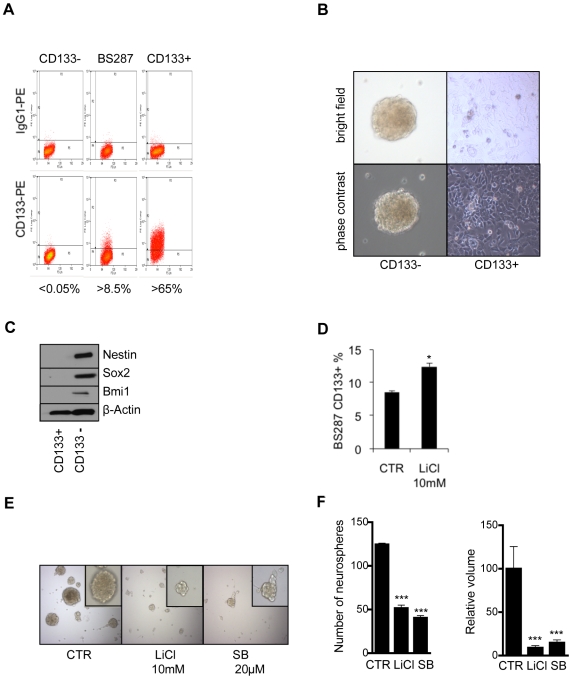
GSK3β inhibition reduced stem cell characteristics in an “*ex vivo*” GBM cell line. (A) CD133 sorting of the BS287 “*ex vivo*” GBM cell line. (B) Growth characteristics of the CD133+ and CD133- populations in BS287. (C) Western blot analysis of Nestin, Sox2, Bmi1 and β-actin in BS287. The CD133- population showed stem cancer-like characteristics. (D) Cells in the CD133+ population with a more differentiated geno/phenotype increased after GSK3β inhibition. (E, F) Inhibition of GSK3β led to a reduction in the number and volume of neurospheres in the BS287 “*ex vivo*” cell line.

It has been argued that GBM cells grown for many passages in standard medium do not mirror the stem cell compartment within the original tumor [38]. To examine this, the “*ex vivo”* cell line BS287 was analyzed which had been isolated from a fresh tumor biopsy and directly grown as neurospheres in neurobasal medium supplemented with bFGF and EGF, thus favoring expansion of cancer stem cells. Interestingly, the population with a stem cell-like signature in this cell line was represented by the CD133- and not by the CD133+ population (Figure 6A–C). Only the CD133− population expressed elevated levels of Nestin, Sox2 and Bmi1 and formed neurospheres (Figure 6B, C). Inhibition of GSK3 decreased the stem cell like (CD133−) population (Figure 6D) and also altered protein levels of stem cell and differentiation markers, mainly decreased Sox2 levels (Figure S3). Induction of differentiation impairs the ability of precursor cells to form neurospheres. Inhibition of GSK3 significantly reduced the number and volume of neurospheres in BS287 cells (Figure 6E, F). The results show that inhibition of GSK3 reduces the cancer stem cell pool and that CD133 may not be a reliable cancer stem cell marker.

### GSK3 inhibition reduces colony formation and induces apoptosis in GBM cells

**Figure 7:**
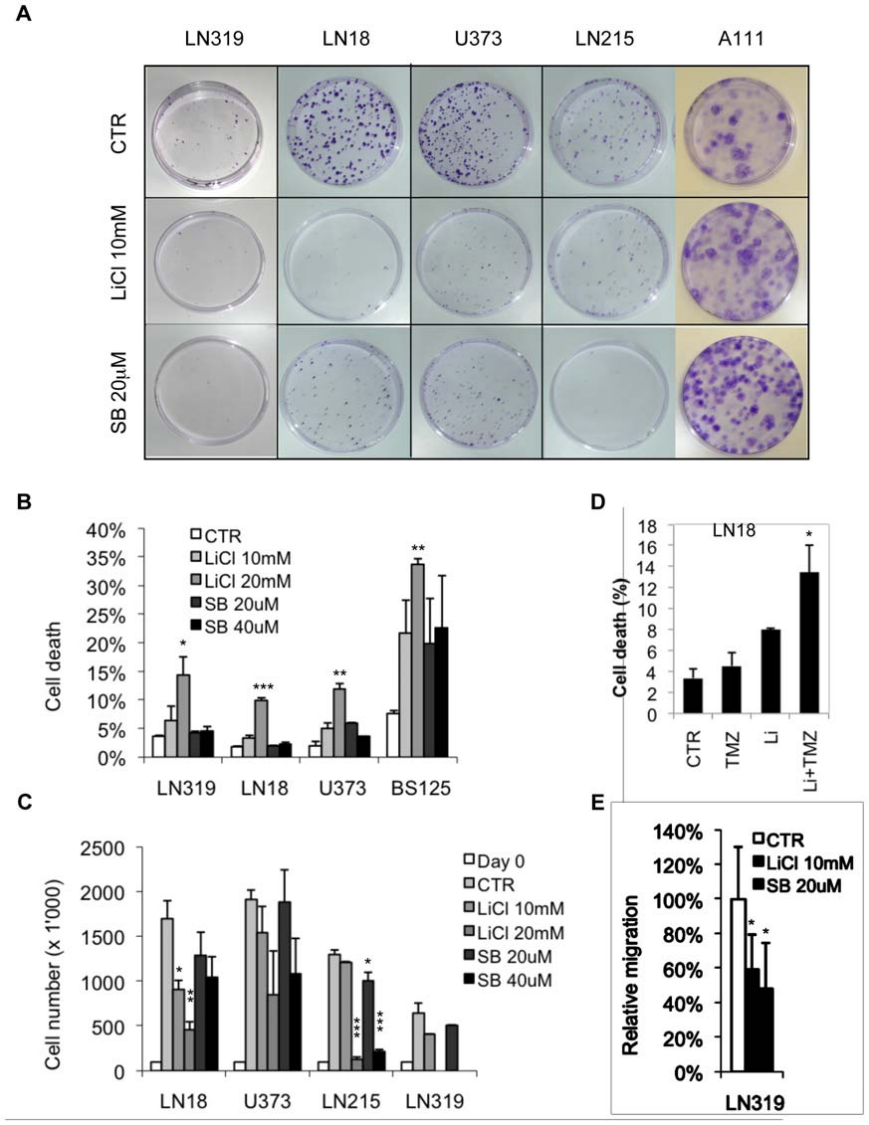
GSK3β inhibition reduced colony formation of GBM cells. (A) A colony formation assay was performed on GBM cell lines treated with 10 mM LiCl or 20 µM SB216763 for 14 days. (B, C) GBM cell lines treated with LiCl or SB216763 for 72 h. (B) Percent cell death and (C) relative cell number relative to the initial seeding. (D) Cell death determined by PI staining of the LN18 cell line after treatment with 10 mM LiCl with or without 50 µg/ml temozolomide (TMZ) for 72 h. *P<0.05, for the combination of LiCl and TMZ compared with each drug alone. (E) GSK3β inhibition significantly reduced migration of the LN319 cell line. (B, C and E) Treated samples were compared to the corresponding control: *P<0.05; **P<0.01; ***P<0.001; one-way ANOVA (Newman-Keuls Multiple Comparison Test).

The effects of GSK3 inhibition on cell proliferation and apoptosis in the GBM cell lines LN18, U373, LN215 and LN319 were analyzed using colony formation, relative cell number and cell death as readout. In a colony formation assay, both the number and size of colonies formed after 14 days of drug treatment were measured. GSK3 inhibitors significantly reduced colony formation in all GBM cell lines tested compared with the untreated control (Figure 7A). The GSK3 inhibitor concentrations used were in the non-toxic range; cell proliferation and survival of the human adipose tissue-derived progenitor cells (A111) were not negatively affected (Figure 7A). GSK3 inhibition by LiCl or SB216763 induced a slight increase in cell death for GBM cell lines LN319, LN18, U373 and BS125 after 72 h (Figure 7B). Induction of cell death was significantly elevated when GSK3 inhibitor LiCl was combined with the standard GBM therapeutic temozolomide in the LN18 cell line (Figure 7D). Cell death was dose-dependent and varied from cell line to cell line. Direct cell counting after exposure of cells to LiCl or SB216763 for 72 h showed inhibition of the proliferation of LN18, LN319, U373 and LN215 cells (Figure 7C). G2-M accumulation was recorded in LN319, LN18, U373 and G2-M arrest in LN215 (Figure S4). In a migration assay, LN319 showed a significant reduction in cell migration following GSK3 inhibition (Figure 7E). These results show that GSK3 strongly reduces colony formation and induces cell death in GBM cell-lines.

## Discussion

Bmi1, a member of the polycomb group proteins, is required for self-renewal of neural stem cells and is upregulated in several cancers. It is also known to repress Ink4a/Arf locus inhibiting progenitor cell proliferation during neural differentiation [39]. In differentiated cells, Bmi1 levels decrease while Ink4a/Arf protein levels increase [40]. As the Ink4a/Arf locus is frequently deleted in brain tumors [41], the role of Bmi1 overexpression in GBM cells appears to be distinct from its repression of the Ink4a/Arf locus. For example, downregulation of Bmi1 did not influence Ink4a/Arf protein levels in tumor cells that retained the Ink4a/Arf locus. Thus, in GBM cells, Bmi1 targets a different pathway. Screening of several key proteins controlling cell cycle, development, metabolism, apoptosis and growth, including Erk, Akt, GSK3β, p16 and p14, Bcl-2, c-Myc, Nestin and Sox2, showed that downregulation of Bmi1 reduced GSK3β protein levels and induced differentiation in cancer cells. In addition, tumor cell proliferation, survival, migration and clonogenicity were markedly reduced.

Discovered some 25 years ago [42], GSK3 has been considered only recently as a therapeutic target for cancer [33]. It has been shown that this enzyme negatively regulates the Wnt, Hedgehog and Notch pathways, which are aberrantly activated in several cancers [33], [43]. This suggests that GSK3 inhibitors could exert a therapeutically negative, pro-survival effect on tumor cells. However, the long-term medical use of the GSK3 inhibitor LiCl for the treatment of psychiatric disorders did not lead to an increase in cancer incidence [44], arguing against an oncogenic effect of GSK3 inhibitors. On the contrary, Cohen *et al*. demonstrated that cancer prevalence in psychiatric patients on long-term LiCl medication was lower than in the general population [44], suggesting even a protective effect of LiCl. The results presented here offer a molecular explanation of this epidemiological observation: administration of LiCl induces differentiation and inhibits proliferation and, thereby, might effectively inhibit tumor formation and progression. Furthermore, the plethora of clinical data on LiCl offer solid information about potential side-effects and it appears safe to assume that normal adult stem cells are not negatively affected, even by long-term use of the drug. The very similar phenotypic and functional alterations induced by either inhibiting GSK3 or by downregulating Bmi1 in the present study points to a functional link between Bmi1 and GSK3. However, further studies are needed to analyze whether there is a direct interaction between Bmi1 and GSK3. Downregulating GSK3 specifically decreased the subpopulation of cancer cells that contained a cancer stem cell-like signature by driving them into differentiation.

Sox2 protein is widely expressed in the early neural plate and early neural tube of several species [45]. In the developing central nervous system, Sox2 expression becomes restricted to the neuroepithelial cells of the ventricular layer, which continue to divide and exhibit an immature phenotype. Cells that leave the ventricular layer lose Sox2 expression [45]. Interestingly, Sox2 has also been implicated in GBM [46], [47] as downregulation of Sox2 reduced cell proliferation and tumorigenicity in GBM cells. Therefore, Sox2 was proposed as a new GBM therapeutic target [46]. At present, inhibitors of Sox2 are not available but the data presented here show that inhibition of GSK3 strongly downregulates Sox2 in GBM cells. This raises the possibility that LiCl or more specific GSK3-inhibitory drugs could be used to decrease the Sox2-dependent tumorigenic potential of GBM cells.

Two main strategies are currently being exploited to eradicate the cancer stem cell (CSC) pool: *i)* chemotherapeutic regimens that specifically drive CSC into apoptosis and thereby deplete the CSC reservoir of the tumor, and *ii)* strategies aiming to drive CSC into differentiation and thereby increase their susceptibility to pro-apoptotic treatments [1]. Given the high degree of drug resistance and the shared cellular and gene expression profiles of adult and cancer stem cells [48], targeting CSC has proven to be difficult. However, induction of differentiation remains a therapeutic strategy for CSC as Piccirillo *et al*. showed that bone morphogenetic proteins can induce differentiation of CD133+ GBM cells, thereby reducing their tumorigenic potential [49]. However, the use of morphogens bears the risk of interfering with the tightly regulated adult stem cell niches. Any strategy to induce differentiation in cancer stem cells must be carefully assessed for any adverse effects on the adult stem cell population.

Our results show GSK3 inhibition to be an attractive strategy for specifically targeting a subpopulation of cancer cells with stem cell-like characteristics. Expression of stem cell and differentiation markers more accurately defined the subpopulation of cells within GBM cell lines and *ex vivo* tumor cells than expression of the CD133 marker. Inactivation of both Bmi1 and GSK3 depleted precursor cells required for tumor maintenance and progression. These data add another facet to the many effects of GSK3 as a regulator of cancer cell identity. Here, GSK3 activation is identified as a key element in maintaining stem cell-like characteristics in a subset of cancer cells, providing these cells with a higher self-renewal capacity. Recently, downregulation of GSK3 was shown to induce apoptosis in glioma cells and to have an anti-migratory effect in glioma sphreoids [50], [51]. The role of GSK3 inhibiton on differentiation was not analyzed. Optimal therapies for cancer aim to spare normal cells with minimal or no general toxicity while depleting malignant cells. The Wnt pathway is involved in regulating cell processes as proliferation, apoptosis, differentiation, mobility and stem cell self-renewal and has been described also as a major regulator of adult neurogenesis in the hippocampus [52]. In the Wnt/β-catenin pathway GSK3β mediates β-catenin degradation. Use of GSK3 inhibitors leads to the accumulation of β-catenin, which then drives cells into proliferation but this effect was not observed in the present study. This may be explained by the constitutive activation of several growth-promoting pathways, such as EGFR and PI3K, commonly found in GBM. This could lead to maximal Wnt signaling target activation masking additional activation. Conversely, differentiation- and apoptosis-inducing programs, which are low in cancer cells, could be influenced by GSK3 inhibition and are, therefore, directly detectable. On the opposite in normal system as in the A111 cells GSK3 inhibition lead to an increased cell proliferation in accordance with the previously described results in neural progenitor cells [53].

shRNA against Bmi1 downregulated not only GSK3 but also Bcl2, Nestin, Sox2 and not p16 and p14 (Figure 2A). The microarray data showed higher levels of GSK3 expression in brain tumors than in normal brain tissue, and protein was found to be expressed in the majority of the tumors analyzed indicating a role for GSK3 in GBM (Figure 2). GSK3 can thus be regarded as an important regulator of tumor cell identity in GBM.

In conclusion, we propose GSK3 inhibitory drugs, e.g. LiCl, as possible first- and/or second-line treatments complementing standard cancer therapy.

The additive effect of combining the GSK3 inhibitor LiCl and the standard GBM therapeutic temozolomide suggests possible sensitization due to the induction of differentiation by interference with GSK3 activity. In addition, the vast clinical experience of this drug with psychiatric patients indicates safe application and the lower cancer prevalence in LiCl-treated patients than in the general population suggests a protective effect of the drug [44]. Clinically, LiCl could be tested in patients receiving standard treatments in an additional therapeutic arm, the clinical hypothesis being that long-term LiCl therapy in stabilized GBM patients may delay tumor recurrence from the residual cancer stem cell pool by driving cancer stem cells into differentiation and apoptosis.

## Supporting Information

Figure S1 Bmi1 down-regulation reduces GSK3β protein levels. (A) GBM cell line U87 is transduced with shRNA against Bmi1 (sh1061) or with scrambled shRNA (scr) (B) Bmi1 down regulation lead to GSK3β reduction in Hs683 using a different shRNA sequence. (C) Bmi1 down-regulation in U87 glioma cell line decreased nestin protein levels, whereas Bmi1 over-expression increased Nestin protein expression.
(2.17 MB TIF)

Figure S2 GSK3β is expressed in primary brain tumors. (A) GSK3β protein expression in a series of primary GBM (1–32). (B) Photomicrograph of the immunohistochemical study showing extensive necrotic areas in GBM compared to Astrocytoma (Hematoxylin-Eosin staining). Arrows point to necrotic areas in GBM.
(3.18 MB TIF)

Figure S3 GSK3 inhibition induces differentiation of the BS287 “ex vivo” cell line. Nestin, Sox2, β-catenin, CNPase, β-tubulin III and β-actin protein expression upon GSK3 inhibition (with LiCl and SB216763 for either 24 or 72 hours) on the BS287 “ex vivo” cell line.
(1.82 MB TIF)

Figure S4 Cell cycle analysis in GBM cell lines treated with LiCl. The GBM cell lines LN319, LN18, U373 and LN215 were treated with 10 or 20 mM LiCl for 24 hours. Percentage of the cells in G1, S and G2-M phase of the cell cycle were evaluated by FACS analysis.
(2.11 MB TIF)
