# Supplementary figures and images for: GSK3β Regulates Differentiation and Growth Arrest in Glioblastoma
Source: PLoS One. 2009 Oct 13;4(10):e7443. doi: 10.1371/journal.pone.0007443 (PMC2757722; doi:10.1371/journal.pone.0007443)

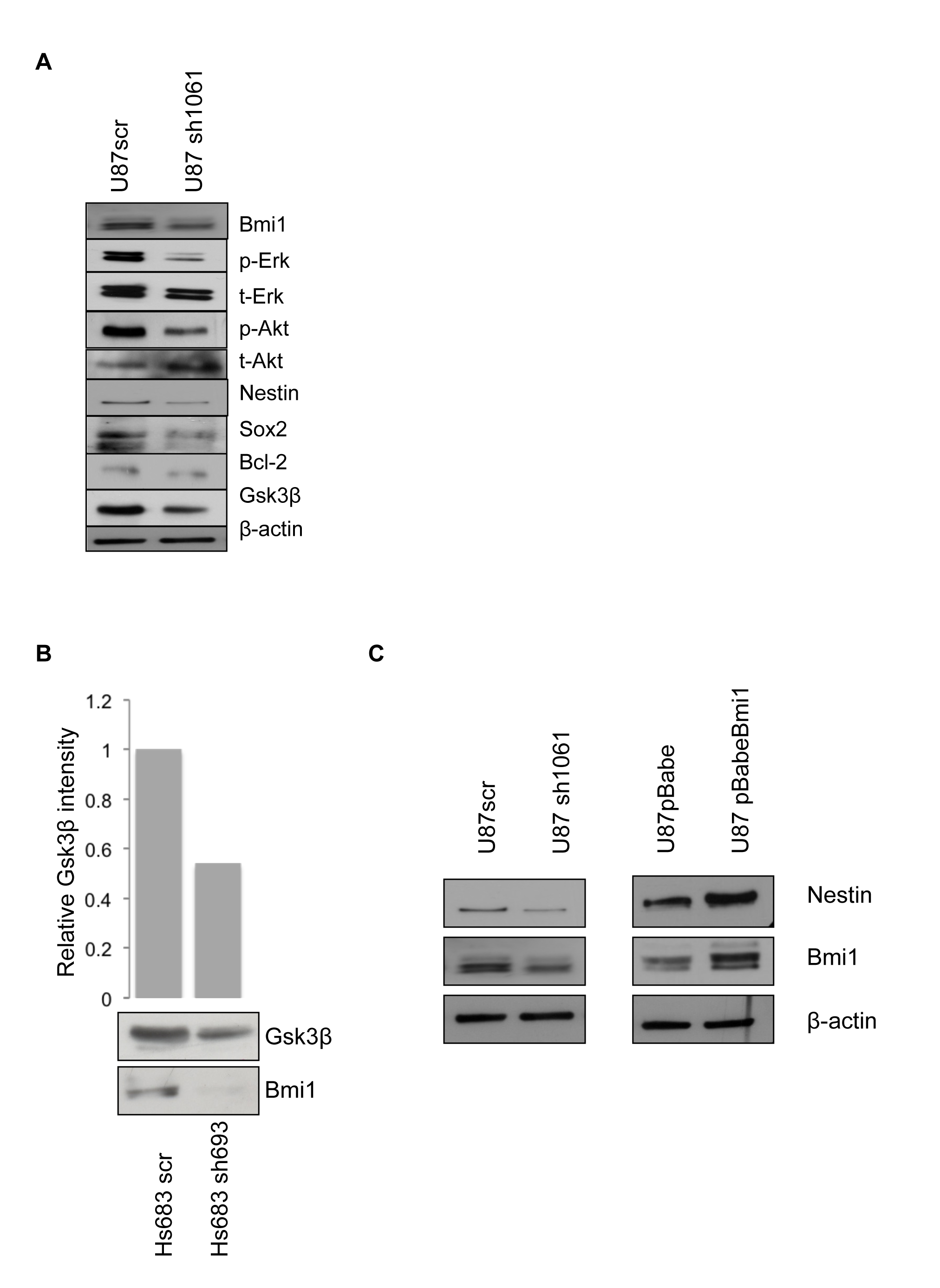

Supplement: Figure S1 — Bmi1 down-regulation reduces GSK3β protein levels. (A) GBM cell line U87 is transduced with shRNA against Bmi1 (sh1061) or with scrambled shRNA (scr) (B) Bmi1 down regulation lead to GSK3β reduction in Hs683 using a different shRNA sequence. (C) Bmi1 down-regulation in U87 glioma cell line decreased nestin protein levels, whereas Bmi1 over-expression increased Nestin protein expression. (2.17 MB TIF) [file pone.0007443.s001.tif]

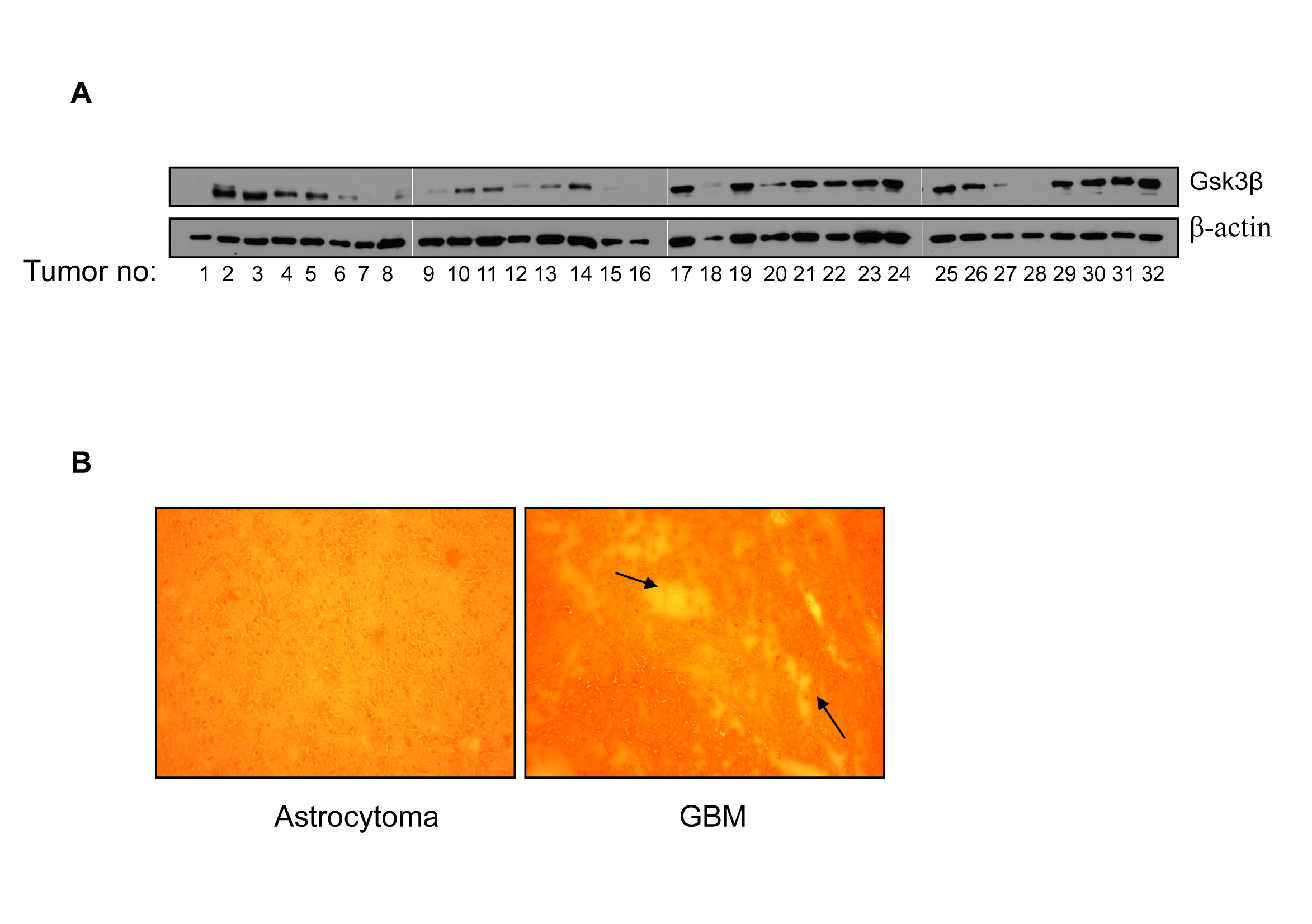

Supplement: Figure S2 — GSK3β is expressed in primary brain tumors. (A) GSK3β protein expression in a series of primary GBM (1–32). (B) Photomicrograph of the immunohistochemical study showing extensive necrotic areas in GBM compared to Astrocytoma (Hematoxylin-Eosin staining). Arrows point to necrotic areas in GBM. (3.18 MB TIF) [file pone.0007443.s002.tif]

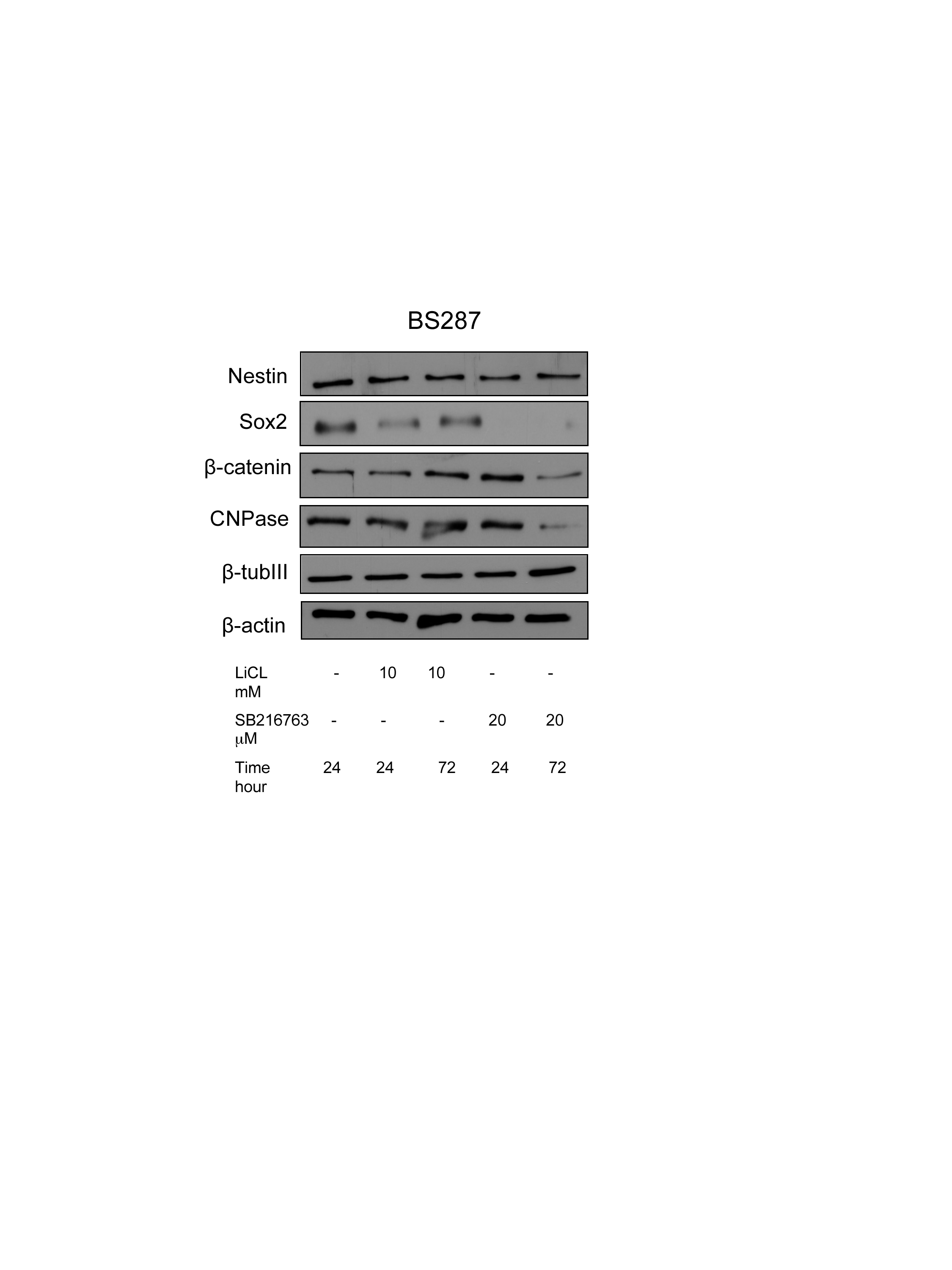

Supplement: Figure S3 — GSK3 inhibition induces differentiation of the BS287 “ex vivo” cell line. Nestin, Sox2, β-catenin, CNPase, β-tubulin III and β-actin protein expression upon GSK3 inhibition (with LiCl and SB216763 for either 24 or 72 hours) on the BS287 “ex vivo” cell line. (1.82 MB TIF) [file pone.0007443.s003.tif]

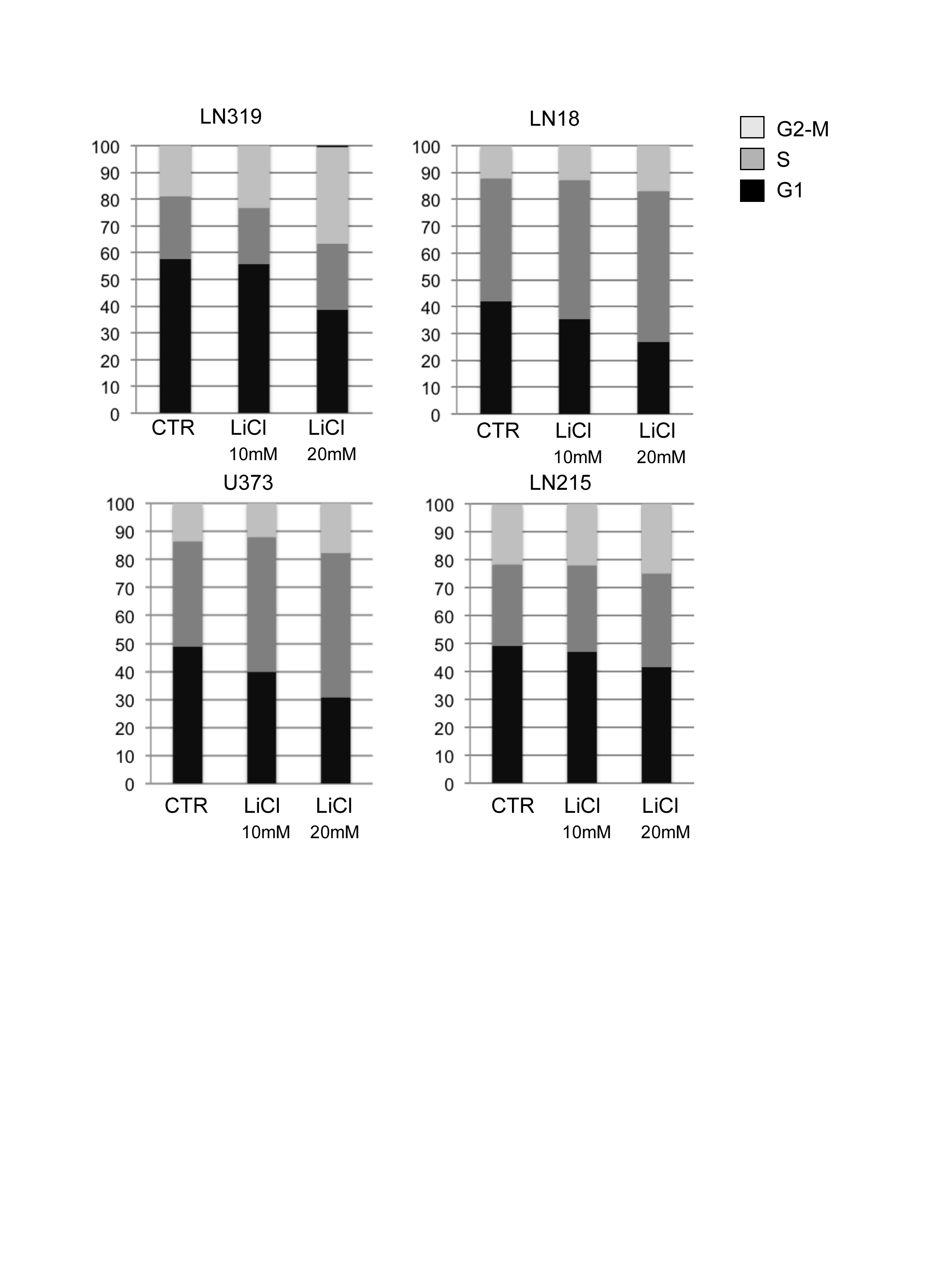

Supplement: Figure S4 — Cell cycle analysis in GBM cell lines treated with LiCl. The GBM cell lines LN319, LN18, U373 and LN215 were treated with 10 or 20 mM LiCl for 24 hours. Percentage of the cells in G1, S and G2-M phase of the cell cycle were evaluated by FACS analysis. (2.11 MB TIF) [file pone.0007443.s004.tif]
